# A bacterial toxin that cleaves Ras oncoprotein

**DOI:** 10.18632/oncotarget.5113

**Published:** 2015-08-07

**Authors:** Marco Biancucci, Karla J.F. Satchell

**Affiliations:** Department of Microbiology-Immuno logy, Feinberg School of Medicine, Northwestern University, Chicago, Illinois, USA

**Keywords:** Ras, MARTX toxin, bacterial toxin endopeptidase, RRSP, Chromosome Section

Rat sarcoma protein (Ras) mediates signal transduction from membrane receptors, such as epidermal growth factor receptor (EGFR), activating signaling cascades leading to phosphorylation of extracellular signal-regulated kinases (ERK1/2). Phosphorylated ERK1/2 activates transcription factors promoting cell proliferation, differentiation, and survival.

Ras is a small guanosine triphosphatase (GTPase) that cycles between the active (GTP-bound) and inactive (GDP-bound) states. The interaction of inactive Ras with guanidine exchanging factor proteins (GEFs), such as SOS, induce large but localized structural changes that destabilize the GDP binding. Since the GTP concentration in the cytoplasm is higher than GDP, Ras becomes GTP-bound, initiating the phosphorylation cascade. GTPase accelerating proteins (GAPs) increase the intrinsic Ras GTP hydrolysis activity by a hundred-fold, cycling Ras back to the GDP-bound state and blocking signal transduction. HRas, NRas, KRas-4A and KRas-4B are the major isoforms in human cells.

Oncogenic mutations in the *Ras* gene impairs the GTPase activity of the Ras protein, leading to its permanent activation and constitutive stimulation of the Ras-ERK pathway. Mutated Ras has been found in 30% of human cancers, but with different distribution among Ras isoforms and cancer types. KRas-4B is mutated in 86% of human cancers, including pancreatic, lung, and colorectal carcinomas, while mutated NRas is almost exclusively found in melanomas [1]. Although several mutations have been demonstrated to impair Ras GTPase activity, residues G12V/C, G13V/D and Q61R are the most recurring Ras cancer mutations. For this reason, Ras is considered one of the most important oncotargets. However, ever since the initial implication of Ras in tumor initiation and progression by Der in 1982 [2], the development of Ras inhibitors has been a major challenge. The micro-molar affinity of Ras for GTP and GDP thwarted design of small molecules able to displace these nucleotides from the binding site. Detailed assessment of the Ras structure revealed there were no clear grooves to target in order to inhibit the interaction with downstream effectors. Although the current knowledge about Ras biochemistry and biology is deep, after more than three decades of research, there are still no approved Ras inhibitors for treatment of cancer.

**Figure 1:**
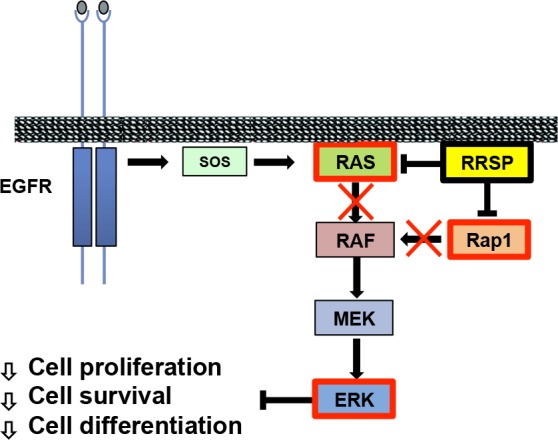
Schematic representation of Ras activation and its downstream signaling Dimerization of EGFR activates Son of Sevenless (SOS), a Ras GEF. SOS exchanges GDP with GTP in Ras nucleotide binding site. Ras-GTP binds to RAF, inducing autophosphorylation and dimerization of RAF and subsequently activating downstream protein kinases MEK and ERK. Processing of Ras and Rap1 by RRSP blocks the phosphorylation cascade, inhibiting cell proliferation, survival and differentiation.

Recently, we initiated study of a naturally-occurring effector domain known as domain of unknown function in 5th position, or DUF5, from the Multifunctional-Autoprocessing-Repeats-in-Toxins (MARTX) toxin of *Vibrio vulnificus*, a bacterial pathogen associated with severe sepsis and necrotizing tissue infections. This toxic effector domain was revealed in our recent paper published in *Nature Communications* to represent a new class of endopeptidase that specifically processes Ras and Rap1 and thus was renamed RRSP for Ras/Rap1 specific peptidase [3]. Specifically, RRSP was found to cleave both Ras and Rap1 between D32 and Y33 within the Switch I region (residues 30-38) [3]. This region is necessary for activation of the GTPases and for their interaction with downstream protein effectors (Figure 1). The implications for bacterial pathogenesis is that loss of Ras and Rap1 signaling would paralyze the immune response normally activated to clear the pathogen.

However, we propose that the discovery of this protein activity will have important implications for cancer treatment. We proved that RRSP not only processes the major Ras isoforms (H, N, and K), but can also process Ras carrying G12, G13 and Q61 oncogenic mutations. In addition, RRSP can be delivered into eukaryotic cells by protective antigen (PA) when fused to the anthrax toxin LF_N_ domain. Moreover, RRSP blocked cell division and proliferation even in colon and breast cancer cell lines carrying the Ras constitutive mutation G13D [3].

We envision that the RRSP effector domain could be developed in the future as new anti-cancer therapeutic agent. In particular, re-engineering PA to selectively target cancer cells could be used to deliver LF_N_ DUF5 into cells to destroy Ras signaling and thereby deregulate tumor growth and proliferation. This approach has already been validated in cell systems in which PA was fused to the epidermal growth factor for delivery of LF_N_-tethered cargo into cancer cells with up-regulated expression of EGFR [4]. Likewise, this system was modified with PA fused to ZHER2 affibody to bind to the HER2 receptor, a membrane protein strongly up-regulated in tumor cells, in particular breast cancer [5]. Although many LF_N_-tethered cargo proteins like diphtheria toxin domain A inhibit protein translation leading to cell death, RRSP would target specifically the Ras pathway, which is up-regulated in many types of human cancer.

As alternatives, RRSP effector domains could be fused or conjugated to specific antibodies to create immunotoxins able to target protein antigens up-regulated on cancer cells. Further, RRSP could be expressed and delivered by bacteria like *Salmonella*, which can deliver therapeutic proteins to solid tumors thereby directly killing cells, inducing apoptosis via signaling pathways, and stimulating the immune system [6]. Moreover, RRSP could be expressed by viruses engineered to specifically infect cancer cells [7].

The ability of RRSP to cleave both normal and mutant forms of Ras indicates that any developed reagent could be successful whether used for Ras cancers, non-Ras cancers, or other Ras-associated diseases. This strategy takes advantage of billions of years of bacterial evolution to create proteins that specifically and potently disable eukaryotic cell pathways that control cell growth, survival, and motility—often the same pathways over activated in tumor cells (Figure 1).
